# A social-ecological examination into the research, policy and health service delivery environment related to early marriage and sexual and gender-based violence among youth in Jordan

**DOI:** 10.1186/s12914-020-00234-y

**Published:** 2020-07-13

**Authors:** Jewel Gausman, Areej Othman, Abeer Dababneh, Maysoon Dabobe, Iqbal Hamad, Insaf Daas, Ana Langer

**Affiliations:** 1 Women and Health Initiative, Department of Global Health and Population, Harvard TH Chan School of Public Health, Boston, MA USA; 2 Maternal and Child Health Nursing Department, School of Nursing, The University of Jordan, Amman, Jordan; 3 Center for Women’s Studies, The University of Jordan, Amman, Jordan; 4 Jordanian Hashemite Fund for Human Development, Amman, Jordan

**Keywords:** Refugees, Youth, Adolescents, Sexual violence, Gender-based violence, Honor killing, Early marriage, Syria, Jordan, Middle East

## Abstract

**Background:**

The determinants of sexual- and gender-based violence (SGBV) and early marriage are embedded across different levels of the social ecological system, including at the individual, family, community, and policy levels. In Jordan and the Middle East, SGBV, honor killing, and early marriage are priority public health and human rights issues that often overlap, and affect a significant percentage of youth. Jordan is home to a large number of refugees from across the Middle East, who may be even more vulnerable to these forms of violence than the local youth. The purpose of this analysis is to 1) synthesize the existing literature and 2) present the perspectives of key stakeholders to identify research gaps, programmatic lessons learned, and opportunities for policy change from an ecological perspective at the individual, community, health-system, and policy/legal levels.

**Methods:**

This study includes 1) a systematic literature review of both published and unpublished literature since 2008 and 2) focus group discussions (FGDs) with key stakeholders representing 18 international and local governmental and non-governmental organizations.

**Results:**

The literature review included 27 documents. Stakeholder discussions highlighted important research and policy gaps. Prevalence estimates of SGBV, honor killing, and early marriage vary across sources; however, all of them indicate that they remain important issues for youth in Jordan. Several sources indicate that early marriage has been increasing in Jordan since the beginning of the war in Syria, especially among Syrian refugees. Refugee youth are particularly vulnerable to SGBV and early marriage given the worsening economic situation in Jordan. The norms, attitudes, and practices that support SGBV in Jordan appear to be reinforced within families and communities. Despite ongoing programs, SGBV services are limited, especially for youth, and there is little awareness of service availability amongst target populations. Laws and policies continue to offer legal justification for SGBV, honor killing, and early marriage.

**Discussion:**

As countries across the Middle East face instability and continue to struggle with the urgent health needs of large refugee and youth populations, this review provides valuable insight relevant to research, programs, and policy in Jordan and across the region.

## Background

Sexual and gender-based violence (SGBV) is a global health and human rights concern with roots in a broad socio-ecological system. SGBV is associated with a wide range of direct and indirect adverse health consequences [1] including, injuries, gynecological disorders, mental health disorders, adverse pregnancy outcomes, and sexually transmitted diseases, high risk sexual behavior, drug and alcohol use, as well as those relating to infant and child morbidity and mortality [2–4]. SGBV is defined as “any act perpetrated against a person’s will and is based on gender-norms and unequal power relationships,” and often encompasses physical, emotional, psychological, or sexual acts [5]. The social ecological model posits that the determinants of SGBV interact and accumulate across the lifespan at multiple levels, including at the individual, family, community, and societal levels [6]. More specifically, the determinants of SGBV may be organized into concentric circles representing an individual’s personal history; the interpersonal and physical context of abuse, such in the home, school, or a relationship; the institutional and social structures in a community, including neighborhoods and peer groups; and the overall political, economic, and social environment [7].

Youth are particularly vulnerable to SGBV, and it ranks as one of the most important health priorities among youth globally [8]. At the individual level, their limited experience with interpersonal relationships and their young age increase their vulnerability [9]. Feelings of shame and fear, combined with institutions that do not acknowledge or are not adequately prepared to support youth victims of SGBV, may reduce youth’s willingness to report SGBV, especially given heightened concerns over breeches in confidentiality and potential repercussion [10]. Attributes of communities, such as poverty and deprivation, can intensify harassment, threats of physical and sexual violence, and increased pressure to engage in early sexual activity, by interacting with and exacerbating pre-existing, gender-based disparities [11], and by limiting the level of social support, community monitoring, and protective services present [12]. Gender-based discrimination is also perpetrated and reinforced through laws and social norms that increase young people’s vulnerability and reinforce the practice of SGBV. Early marriage, for example, is a human rights violation that is rooted in gender inequality that makes young women more susceptible to other forms of SGBV [13], while increasing their vulnerability to a wide range of adverse health outcomes, including those related to physical, mental, sexual and reproductive, and child health.

SGBV in Jordan is relatively widespread [14]. Approximately one in four women have experienced some form of violence from someone within their home (including fathers, husbands, and brothers) [15]. Some populations within Jordan may fare even worse; one study estimated the overall prevalence of violence perpetrated against pregnant women in a Bedouin community at 40.6% [16]. Physical, emotional, psychological and verbal abuse, controlling behavior, and intimidation are often perpetrated against women by their fathers, family members, intimate partners, and other male community members. Youth in Jordan are particularly vulnerable to SGBV because of gender discrimination, secrecy, rigid social expectations, increasing economic hardship and poverty, and a lack of protective institutions. Early marriage and honor killing are specific forms of gender-based violence that are perpetrated against young women in Jordan. Discriminatory laws codify their continued practice, such as by allowing for loopholes that enable families to marry girls below the age of 18 years on religious or social grounds [17], maintaining legal justification for honor killing [18], and continuing practices that introduce gender bias in official statistics so that instances of honor killings are not reliably recorded [19]. Estimates from the early 2000s indicate that per capita, Jordan may have had some of the highest rates of honor killing in the world [19].

Recently, the ongoing conflict in Syria and instability elsewhere in the region, has caused youth in Jordan to become increasingly vulnerable to SGBV [20, 21]. Jordan is currently home to at least 1.2 million Syrian refugees, two million Palestinian refugees, and 500,000 Iraqi refugees [22–24]. SGBV is prevalent across the Middle East, and its prevalence is often exacerbated in times of conflict and displacement [25]. For many refugees in Jordan, institutionalized discrimination in their country of origin has increased women’s vulnerability to SGBV during displacement by limiting their self-sufficiency and their ability to pursue education and employment opportunities [26]. Syrian refugees have disclosed extremely high rates of SGBV to humanitarian workers, and some female refugees have reported having to engage in forced sexual acts in order to ensure that they have the necessities to survive [27]. Rape, prostitution, and forced marriages have been described as common in Jordanian refugee camps, while services for survivors are almost non-existent [28]. Female refugees face greater obstacles in obtaining legal status, protection, and resettlement [29], thus subjecting them to prolonged periods of vulnerability. Exposure to environmental stressors, such as lack of employment, security, and essential services, has been linked with increases in family violence [30]. Last, some reports indicate increased tension between refugee and host communities [28], with backlash increasing the vulnerability of the most disadvantaged refugees.

To date, there have been limited efforts to consolidate research and programmatic experience on SGBV and early marriage among Jordanian and refugee youth living in Jordan. This analysis 1) synthesizes the existing literature and 2) presents the perspectives of key stakeholders to identify research gaps, programmatic lessons learned, and opportunities for policy change from an ecological perspective at the individual, community, health-system, and policy/legal levels. The goal of this study is to provide useful information that is not only relevant to the Jordanian context, but in countries throughout the Middle East that face similar issues and displaced populations.

## Methods

The methodology used in this study includes 1) a systematic literature review of both published and unpublished literature, including research, reports, and policy documents, and 2) focus group discussions (FGDs) with key stakeholders. This research was approved by both the Harvard T. H Chan School of Public Health Institutional Review Board (IRB) and the University of Jordan School of Nursing IRB. Informed consent was collected from all participants in accordance with approved ethical procedures.

### Literature review

Several search strategies were used to obtain the literature analyzed in this review. Academic publications were obtained through structured searches on PubMed, JSTOR, Embase, Medline, Web of Science, CINAHL, and Google Scholar. The search terms utilized included combinations of the following terms: *gender-based violence, sexual violence, violence, abuse, harassment, honor killing, child or early marriage, adolescent marriage, and Jordan.*

Non-academic literature was obtained using four approaches. Searches of gray literature databases, including OpenGrey, PopLine, USAID Development Experience Clearing House, and Knowledge4Health were conducted using the search terms identified for the academic literature search. Furthermore, internet searches and reviews of key organization’s websites were also performed. Last, organizations that were identified through consultations with local experts were contacted individually to obtain any relevant documents available only in hard-copy.

Documents were included in the review if they 1) focused on youth between the ages of 10–24 years, 2) focused on SGBV, 3) were published after the year 2008 in Arabic or English, and 4) focused on Jordan. The literature review was conducted in 2018, thus, the documents pertaining to the previous ten years were included in the study so as be most relevant to the current programmatic context. Two individuals screened and reviewed abstracts or executive summaries from each document obtained in the search using the eligibility criteria identified above. Consensus was reached to identify a preliminary list of relevant documents. Full text was obtained for each of the documents on the preliminary list and eligibility was again reviewed. Results were then extracted from each eligible document.

### Focus group discussions with key stakeholders

Four FGDs were conducted with stakeholders representing 18 local and international organizations from August to September 2018. FGDs were organized according to similarity of each organization’s role and mandate. Organizations were identified through consultations with local experts on the issues of interest and snowball sampling. Participants from each organization were selected based on expert recommendation, willingness to participate, and expertise and experience with topics related to youth, refugees, violence, and women’s health.

Stakeholders included representatives from one international donor organization, two United Nations (UN) agencies focused on youth and refugee issues, seven local governmental entities focused on health, youth, and population issues, and eight local and international non-governmental organizations. Some organizations sent more than one participant for a total of 22 participants. Participants were asked about their organization’s past programmatic experience, and their perspectives on existing policies, barriers and challenges in the domains of SGBV and early marriage. Additionally, stakeholders were asked to provide their recommendations to strengthen existing policy and programs.

### Data analysis

The social ecological model guided the analysis [31] which enabled an analysis of research, programs, and policy targeting different levels of the contextual environment pertinent to youth. Data from publications were abstracted and synthesized as being either directly focused on youth, or focused on other ecological levels, such as family, schools, and the service delivery, policy, and legal environments. FGDs were then conducted as an iterative step with the dual purpose of filling some of the gaps identified in the literature review and building upon the themes that emerged from across the different levels of the social ecological model. FGDs were recorded, transcribed, and thematically coded by two coders from the research team (one Jordanian and one international), according to codes developed a priori based on the interview guides to correspond to different levels of the ecological model (individuals, parents, communities, service environment, or policy/legal environment,) type of SGBV, and specific underlying determinants (i.e. security, gender, economic, social norms, cultural practices, etc.).

## Results

**Fig. 1:**
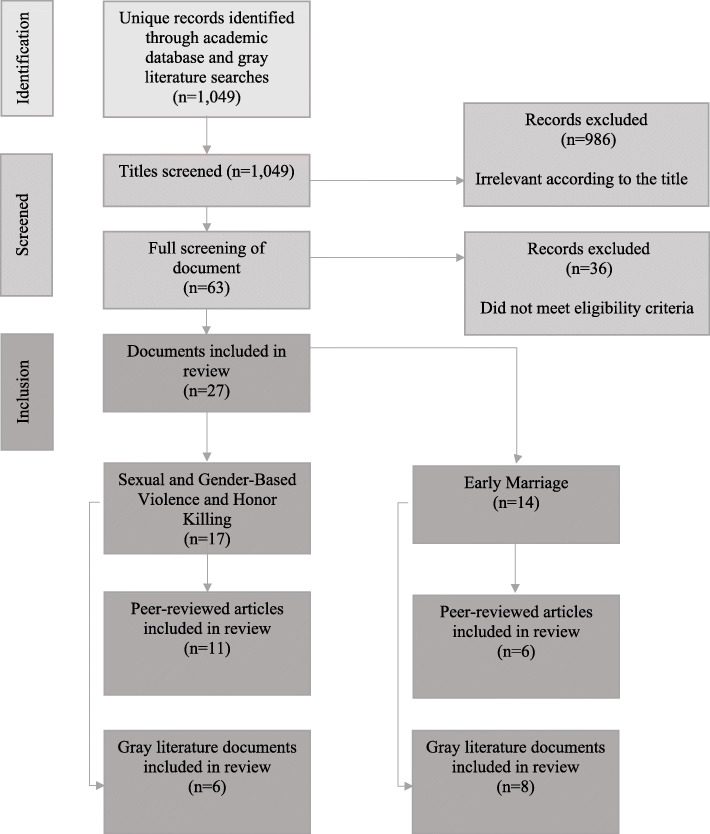
Document identification and screening process in peer-reviewed and gray literature review of youth family planning and reproductive health in Jordan

A total of 1049 documents were obtained in the initial literature search. Of these, 27 documents met the eligibility requirements and were included in the review (Fig. 1**)**. Several of the documents addressed multiple topics. Seventeen documents were found to focus on SGBV among youth populations, 11 of which were obtained in the academic literature and seven were obtained from the gray literature. Within these documents, 15 addressed youth specifically and three addressed the service delivery environment. Four of the documents obtained discussed honor killing. Fourteen documents were found that focus on early marriage in Jordan, and all but six of the documents were retrieved from the gray literature. Of the 14 documents retrieved, 11 focused on youth specifically, and 10 included discussion of the overall policy and program environment. The vast majority of research and documentation related to early marriage in Jordan relates to Syrian refugees. Table 1 provides the list of the documents included in the analysis along with a summary of the methodologies used and key results.

**Table 1:** Summary of the 27 documents included in the analysis according to ecological level and topic of focus pertinent to sexual- and gender-based violence and early marriage among youth in Jordan

Author	Year	Title	Ecological Level	Topic of Focus	Study Design/Description	Key Results
Abedr-Rahman, H; Salameh, HO; Salameh, RJ; Alabdallat, LI; Al-Abdallat, IM [32]	2017	Role of forensic medicine in evaluating non-fatal physical violence against women by their husbands in Jordan	Youth	SGBV	Retrospective analysis of 158 cases of partner assault	No age disaggregated results, but a majority of the sample (46%) was between the ages of 18–28 years of age.
Al-Gharaibeh, Fakir [33]	2016	Debating the role of custom, religion and law in ‘honour’ crimes: implications for social work	Supportive Environment	SGBV - Honor Killing	Twenty-four Jordanian criminal trials of perpetrators of ‘honour’ killings from 2010 to 2014 were analysed.	Most victims were unmarried women or young girls who were murdered by male relatives, usually brothers, for reasons including absence from home, being raped and talking on the phone
Al-Hawari, H; El-Banna, A [34]	2017	A medicolegal study of domestic violence in south region of Jordan	Youth	SGBV	Examination of medical records after reported cases of abuse in population aged between 6 and 18 and above	Young women between the ages of 12–18 were more likely than other age groups to experience sexual abuse. “illegal” pregnancy occurred in 13% of the cases. Other outcomes included young women leaving the home (20%).
Al-Modallal, H; Abu Zayed, I; Abujilban, S; Shehab, T; Atoum, M [35]	2015	Prevalence of intimate partner violence among women visiting health care centers in Palestine refugee camps in Jordan	Youth	SGBV	Convenience sample of 198 Palestinian refugees living in Jordan. 8.7% of the sample was less than 20 years old, 44% of the sample was between 21 and 30 years of age.	Overall, 78% of respondents were victim to at least one form of IPV. 83% of women aged < 20 and 75% of women aged between 21 and 30 experienced control IPV, 63% of women < 20 and 59% of women aged 21–30 experienced economic IPV and 40% of women < 20 and 53% of women 21–30 experienced emotional IPV. Sexual and physical IPV were less common (21% of women, and 27.5% of women experienced physical IPV)
Clark, CJ Spencer RA; Khalaf, IA; Gilbert, L; El-Bassel, N; Silverman, JG; Raj, A [36].	2017	The influence of family violence and child marriage on unmet need for family planning in Jordan	Youth	Early Marriage and SGBV	Cross-sectional study using 2012 DHS	Women from poorer backgrounds and women with lower levels of education are more likely to be married before the age of 18. Twenty two percent (*n* = 1325) of study participants reported IPV and participants who married before age 18 years were more likely to report IPV (27.6%, *N* = 351) compared to those marrying at or after reaching the age of majority (21.0%, *N* = 974). The compounding effect of FV and IPV on unmet need among women who marry as minors suggests the need to strengthen laws against child marriage and to empower girls at risk of early marriage.
Committee on the Rights of the Child, UNHCR [37]	2014	Committee on Rights of Child examines reports of Jordan on the Convention, Children in armed conflict and the Sale of children	Supportive Environment	Early marriage and SGBV (honor killing)	Not Applicable	Description of laws surrounding early marriage in Jordan. Prevalence of honor killings.
Committee on the Rights of the Child, UNHCR [38]	2013	Consideration of reports submitted by States parties under article 44 of the Convention Combined fourth and fifth periodic reports of States parties due in 2011 Jordan	Supportive Environment	Early marriage and SGBV (honor killing)	Not applicable	Description of laws surrounding early marriage in Jordan.
Doedens, W; Giga, N; Krause, S; Onyango, MA; Sami, S; Stone, E; Tomczyk, B; Williams, H [39]	2013	Reproductive health services for Syrian refugees in Zaatri Refugee Camp and Irbid City Jordan: an evaluation of the Minimum Initial Service Package March 17–222,013	Supportive Environment	Early Marriage	Interviews with female youth (18–24 years of age)Health facility assessmentsKey informant interviews	Advocacy on child marriage/trafficking is not useful or informed and has affected capacity to address other issues because of sensitivities and its impact on Syrians by causing backlash Some foreign men have come into camps looking for girls to marry. The participants in Zaatri and Irbid noted that the most common age to marry was approximately 15, with a range from 13 to 20, depending on the area of Syria in which they had previously resided. In Irbid, women described reasons such as fathers wanting someone to protect their daughters from bad security in the camp or not having responsibility in the situation of their daughters being raped.
Eisner, M; Ghuneim, L [13]	2013	Honor killing attitudes amongst adolescents in Amman, Jordan	Youth	SGBV -Honor Killing	Cross sectional survey with 856 adolescents aged 14–16 recruited from schools in Jordan	Descriptive findings suggest that about 40% of boys and 20% of girls believe that killing a daughter, sister or wife who has dishonored the family can be justified. Being of low SES and education is associated with more support for honor killing
Fowler, R [40]	2014	Syrian Refugee Families’ Awareness of the Health Risks of Child Marriage and What Organizations Offer or Plan in order to Raise Awareness	Supportive Environment	Early Marriage	Qualitative interviews conducted with: 4 Syrian families at health center, 2 physicians, and 2 staff from UNICEF and UNFPA	Syrian refugees’ perception of early marriage depends on their situation in Jordan, such as their ability to provide for their family, their feeling of safety, and their access to services like health and education. Provides description UNICEF UNFPA programs
Higher Population Council [41]	2017	A study of early marriage in Jordan	Youth	Early Marriage	Analyzes data from Jordan Department of Statistics	Presents data showing increase in early marriages among Syrians and rates among Jordanians and other refugee groups. Early marriages among Syrians rose from 33.2% in 2010 and 43.7% in 2015
Jordan Communication Advocacy and Policy Activity [42]	2016	Family Planning Among Syrian Refugees Living in Jordan	Youth	SGBV	Review of assessments and surveys from 2013 to 2016 focused on family planning among Syrian women in Jordan	Wife beating was acceptable to some married and unmarried male youth in several instances Conflict in Syria and displacement have made women more susceptible to GBV, especially girls
Jordan Communication Advocacy and Policy Activity [42]	2016	Exploring Gender Norms and Family Planning in Jordan: A Qualitative Study	Youth	Early Marriage	Focus group discussions conducted with 408 participants, including unmarried male and female youth between the ages of 18–24	One-third of the married Jordanian men and one-fifth of unmarried Jordanian male youth agreed that violence was appropriate if woman refused to bear children or used family planning without permission. Half of unmarried Jordanian and Syrian males and females reported that violence against women was unacceptable, even if limited to shouting or threatening
Jordan Communication Advocacy and Policy Activity [43]	2015	Knowledge attitudes and practices toward family planning and reproductive health among married women of reproductive age in selected districts in Jordan	Youth	SBGV	Survey of 2040 married women of reproductive age in 8 districts in Jordan	65% of girls between the ages of 15–19 agreed that a woman should tolerate verbal, physical, or sexual violence to keep family together. 92% of girls aged 15–19 support at least one reason given for husband to beat wife.
Jordan Health Communication Partnership [44]	2012	Evaluation of the Arab Women Speak Out (AWSO) Initiative - 2nd Tier (Phase II) - in Irbid Governorate, Jordan, 2012	Youth	Early Marriage	Evaluation of an intervention to raise awareness related to early marriage	85% of all recipients disagreed that “early marriage is a good basis for a happy and stable life.” 90% of all participants reported that they intend to discourage or have already discouraged their daughters or nieces from marrying before the age of 18
Lenze J; Klasen; S [45]	2017	Does Women’s Labor Force Participation Reduce Domestic Violence? Evidence from Jordan	Youth	SGBV	Secondary analysis of 2012 Jordan Population and Family Health Survey	About 25% of married women aged 15–29 experience domestic violence; no other age disaggregated results
Okour, A; Raja, B [16]	2011	Spousal violence against pregnant women from a Bedouin community in Jordan	Youth	SGBV	A cross-sectional survey with 303 pregnant women who attended antenatal clinics in Al-Mafraq, northern desert of Jordan	Overall prevalence of violence was 40.6% during pregnancy. Included women aged less than 20 (but very small sample) and women between age 20–29; no significant association was found with regard to age.
Oweis, A; Gharaibeh, M; Alhourani, R [15].	2010	Prevalence of violence during pregnancy: findings from a Jordanian survey	Youth	SGBV	A convenience sample of 316 pregnant women was recruited from five Maternal and Child Health Centers, located in Irbid City in the North of Jordan	Women between the ages of 15–24 are included in the sample overall, but no disaggregated results for youth. One finding: age is not associated with the prevalence of violence therefore authors conclude that violence occurs frequently throughout marriage. The prevalence of physical, emotional, verbal and sexual violence by husbands during pregnancy was 10.4, 23.4, 23.7, and 5.7%, respectively.
Safadi, R; Swigart, V; Hamdan-Mansour, AM; Banimustafa, R; Constantino, RE [46]	2013	An Ethnographic–Feminist Study of Jordanian Women’s Experiences of Domestic Violence and Process of Resolution	Youth	SGBV	Narrative life histories regarding women’s experiences with violence. Women between the ages of 22 and 24 years were included in the sample	violence and discrimination often begins in childhood, perpetrated by their fathers. Fathers encouraged the reproduction of abusive behavior and established discriminatory beliefs towards women by their fathers. of the women described being forced into early marriage by controlling fathers and raped by future spouses
Sahbani, S; Al-Khateeb, M; Hikmat, R [47]	2016	Early marriage and pregnancy among Syrian adolescent girls in Jordan; do they have a choice?	Youth	Early Marriage	Commentary	The percentage of underage Syrian refugee girls who registered their marriages in Jordan increased three times from 2011 rates, to reach 32% of all marriages by 2014.
Samari, G [28]	2017	Syrian Refugee Women’s Health in Lebanon, Turkey, and Jordan and Recommendations for Improved Practice	Youth; Supportive Environment	Early Marriage and SGBV	Review article and synthesis of assessments between 2011 and 2016 focused on Syrian women’s health in Jordan	Need to integrate mental health and sexual and reproductive health services In camps, prostitution, rape, and forced underage marriages are very common In 2013, 25% of Syrian refugee girls were married under the age of 18, and continues at this rate. There has been fatwas issues against this practice, but seems of little use. In 2014, the proportion of deliveries in girls under the age of 18 was 11%. No policy in Jordan handles gender-based violence or care for survivors of sexual violence Describes existing UNFPA and Government of Jordan SGBV initiatives.
Save the Children [48]	2014	Too young to wed: The growing problem of child marriage among Syrian girls in Jordan.	Youth; Supportive Environment	Early Marriage	Results from desk review and qualitative interviews	Examines reasons for early marriage among Syrians in refugee settings: gender inequality, violence, poverty, and rape, and also impact on girls: forced to marry older men, cannot return to school, pregnancy, and lack of social protection as marriages are often unregistered.
Sexual and Gender-Based Violence Sub-Working Group [49]	2015	Sexual and Gender Based Violence Refugees in Jordan	Youth	SGBV	Description of UNFPA’s Safe Spaces program implemented among women and girls in camps	In Jordan, UNFPA’s supported safe spaces are closely linked with Sexual and Reproductive Health services. They are either present in the same physical space or are carried out by the same partners to facilitate referral.
Smith, JR; Ho, LS; Langston, A; Mankani, N; Shivshanker, A; Perera, D [50].	2013	Clinical care for sexual assault survivors multimedia training: a mixed-methods study of effect on healthcare providers’ attitudes, knowledge, confidence, and practice in humanitarian settings	Supportive Environment	SGBV	Survey of 14 health service providers in Jordan working in refugee settings	Providers in Jordan demonstrated very limited past experience with sexual assault
Spencer, D [51]	2015	To protect her honour. Child marriage in emergencies-the fatal confusion between protecting girls and sexual violence	Youth	Early Marriage	Focus group discussions	Early marriage was seen as a form of ‘protection,’ benefitted families by reducing the total number of people in a household. Girls begin to feel rejected by their families around age 20, and some experience emotional abuse Traditional processes to verify husbands no longer exists in displacement, increases risk of SGBV. Negative view of Syrians by Jordanians – increased harassment of Syrian girls Description of initiatives to counter child marriage
UN Women [52]	2013	Gender-based Violence and child Protection among Syrian refugees in Jordan, with a focus on early marriage	Youth; Supportive Environment	Early Marriage and SGBV	in-depth interviews with key community informants, FGDs with married and unmarried Syrian refugees of both sexes and different survey questionnaires given to 613 individuals.	Data not disaggregated by age: Family members are often the first choice for support in the case of SGBV. Only 3.9% of women said that they would go to a heath center in the case of sexual violence. Most women said they should stay silent and that there is no where to go in their community. 83% did not know services were available in their community for SGBV. Half of respondents indicated that the normal age for marriage was under the age of 18 years. Most believed the age of marriage had decreased for boys and girls since the war. Economic pressure was an important reason for marriage. Of the families reporting that bride price was one of the top three sources of income, 80% were in the north of Jordan.
UNICEF [53]	2014	A study on early marriage in Jordan 2014	Youth; Supportive Environment	Early Marriage	Qualitative and quantitative analysis Interviews and focus group discussions were conducted in 2013 with mothers, fathers, women who had married early, NGO and government service providers, judges, religious and community leaders. Quantitative data from the Department of the Chief Justice	Palestinian, Iraqi and Syrian refugees in Jordan all report economic factors as an important determinant of child marriage. Maintenance of tradition also important. Syrian crisis has added to the number of child marriages in Jordan among Syrian refugees
UNICEF [54]	2013	Shattered Lives: Challenges and priorities for Syrian children and women in Jordan	Youth; Supportive Environment	Early Marriage and SGBV	27 key informant interviews, six focus group discussions, and a Safety Audit	Increased domestic violence, especially against adolescent girls, boys and women Heightened fear of sexual harassment and sexual violence among girls and women Women and girls aged 12–18 are the most likely targets for domestic violence, but boys are also affected. Male spouses and male parents/caregivers are considered the main perpetrators Prevalence of child marriage unknown in Zaatri camp, but anecdotal evidence suggests spousal age gap is increasing, and bail out system is incentivizes early marriage as economic situation worsens.

The results are organized to correspond to the different levels of the social ecological model. In the first section, research on the prevalence of SGBV, honor killing and early marriage within various population groups in Jordan, as well as individual-level determinants, is discussed. The remaining sections focus on the level of families and communities, the service delivery environment, and the policy environment. Results from the FGDs are integrated into each section.

### Prevalence of SGBV, honor killing and early marriage among youth in Jordan

Eight of the documents that were included in the review provided estimates of the prevalence of specific types of gender-based violence across youth population subgroups. These documents indicate that violence is commonly experienced by young women, and across the life-cycle. A study that uses data from the 2012 Jordan Population and Family Health Survey (JPFHS) suggest that about 25% of married women aged 15–29 experience domestic violence, which is similar to that estimated among all women of reproductive age [45], while another study focused on SGBV during pregnancy found no differences in prevalence according to age and found that across all ages, physical, emotional, verbal and sexual violence perpetrated by husbands during their wife’s pregnancies were estimated to be 10.4, 23.4, 23.7, and 5.7%, respectively. A forensic analysis of medical records of reported cases of abuse among Jordanians found that in the Southern region of Jordan young women between the ages of 12–18 were more likely than other age categories to report and seek medical attention for having experienced abuse (32.8% of the 128 cases reviewed occurred among women aged 12–18), and sexual abuse was the most common form (41.1% of all cases, but not disaggregated by age) [34]. The consequences of sexual abuse were severe: 20% of the young women left their home and 13% of the victims became pregnant with pregnancies that are considered to be illegal by the state, as they were outside of wedlock [34]. Another form of violence that youth experience in Jordan is family violence. A study using data from the 2012 Jordan Population and Health Survey found that family violence after the age of 15 years was commonly reported among young women (14.7% of women who were married under the age of 18 and 17.2% who were married after the age of 18) [36].

SGBV among Syrian and Palestinian refugees is also relatively common. Among Syrian refugees, the five documents retrieved focused on refugees living within formal camp settings. According to a qualitative study among adolescent girls in Zaatri camp, young women aged 12–18 years are the most common targets of domestic violence, and young women reported an increase in domestic violence since coming to Jordan [54]. Male spouses and fathers/male guardians are thought to be the most common perpetrators of violence against women [54]. Many Syrian girls, boys and women also experience sexual harassment and feel threatened by sexual violence; according to one document, girls and women identify their homes (as a result of their limited physical security, such as the absence of locks on their homes, the presence of male relatives and the lack of privacy), showers/latrines, and communal areas (kitchens) as unsafe places within camps [49]. Existing data also suggest that rape and sexual violence are likely to be the most common types of violence experienced by young Syrian women and girls living in camps [43]. The results of one study mention that young men also feel vulnerable to sexual assault and rape [52].

Only one study was retrieved focused on SGBV among Palestinian refugees [35]. The study used a cross-sectional convenience sample of women attending UNRWA clinics and found that 83% of women less than 20 years of age and 75% of women aged between 21 and 30 years experienced interpersonal violence related to controlling behavior; 63% of women ages less than 20 and 59% of women aged 21–30 years experienced economic interpersonal violence; and 40% of women aged less than 20 years and 53% of women 21–30 years experienced emotional interpersonal violence [35]. Sexual and physical interpersonal violence, while still very frequent, were less commonly reported (21% of women aged less than 20 years, and 27.5% of women aged between 21 and 30 years reported experienced physical interpersonal violence) [35].

The prevalence of honor-killing in Jordan is not well documented. The 2014 Jordanian government submission to the UN Committee on the Rights of the Child (UN CRC 2014) identified 50 cases of honor killing between 2000 and 2010. Young women are most likely to be victims of honor killings. The report showed that 12% of victims were aged under 18 years, and 56% were aged 18–28 years [33]. Among these victims, 25% were killed for spending the night away from home; 21% were killed because of illegitimate pregnancy, 13% were killed because they were raped, and 1% were killed because of talking to someone on the phone [33]. In these cases, rumors are considered sufficient to justify honor killing [33].

The prevalence of early marriage in Jordan remains a contentious issue among all population subgroups, and the data often differs by source. One study that examined the marriages registered by Shari’a courts in Jordan found that 13.2% of marriages included a woman under the age of 18; 12.7% among Jordanians, 17.6% among Palestinians, 25% among Syrians, and 4% among Iraqis in 2013 [55]. In another study conducted in 2014, data from the General Population and Housing Census indicated that 32% of Syrian marriages involved a girl between the ages of 15–17 [17]. In focus group discussions in Zaatri and Irbid, participants mentioned that the most common age for girls to marry in their communities was approximately 15, with a range from 13 to 20, and the age depended on where the family originally resided in Syria [39]. An analysis using data from the 2012 JPFHS suggests that women from poorer backgrounds and women with lower levels of education are more likely to be married before the age of 18 when compared to richer and better education women [36]. An analysis using data from the Jordan Population and Family Census also found similar percentages of girls who were married before the age of 18 years. This study found an increase in the percent of marriages to girls under the age of 18 years, from 13.7% in 2011 to 18.1% in 2015. The governorates with the highest percentages of marriages occurring to girls under the age of 18 were Mafraq (24.5%), Zarqa (18.8%) and Irbid (17.7%) [41].

In Jordan, it is believed that early marriage in the Syrian population has increased dramatically since the start of the conflict. One study conducted among Syrian youth indicates that according to young women themselves, early marriage has become much more common since the beginning of the war [48]. In 2011, the percentage of registered Syrian marriages involving a girl between the ages of 15–17 was estimated at 12 to 13% [43, 56]. An analysis of data from the Population and Family Census found that the total percentage of registered marriages of girls under the age of 18 years among Syrians in Jordan increased from 12% in 2011 to 34.6% in 2015 [41]. Within these marriages, the spousal age gap has also increased. In a qualitative study, Syrian youth indicated that in Syria, young women who were married early were often also married to a young man of a similar age; however, in Jordan, youth indicate that it is much more common for young girls to marry men who are much older, as an older man is thought to be more capable of offering protection [52]. In 2012, one study reported that among Syrian girls married between the ages of 15–16, 16.2% married men who were 15 or more years older, 31.8% married men who were 10–14 years older, and 37.2% married men who were 5–9 years older [53]. Interestingly, a much lower percentage of Palestinian and Jordanian girls who marry early marry someone much older than them, only 6% of Palestinian girls and 7% of Jordanian girls who married early married someone more than 15 years older than them [43].

The increasing prevalence of early marriage in Jordan may put a larger number of young women at risk of SGBV within their marriages. Many of the victims of early marriage find themselves in exploitative and abusive situations, and at increased risk of sexual and gender-based violence [56]. One study using data from the 2012 JPFHS found that among all married women of reproductive age in Jordan, those who married before the age of 18 were more likely to report experiencing intimate partner violence compared to those who married at or after the age of 18 [36]. Among Syrians, qualitative research suggests that young girls may be at increased risk of SGBV within their marriage, which is partially attributed to the breakdown in family processes that verified the suitability of the groom as a result of the war and displacement [43, 51].

The studies retrieved discussed the challenges in estimating the prevalence of SGBV and early marriage among Jordanian and refugee youth in Jordan. Both Jordanian and Syrian social norms emphasize the importance of privacy regarding events that take place within the home, and most cases of gender-based violence go unreported to authorities [54, 57]. Syrian women rarely report abuse; the results of one study indicate that a woman would face additional threats or violence from family members if she were to report violence to authorities [52]. Additionally, one report indicated that lack of freedom of mobility among women (and young women in particular) may also limit a woman’s ability to report situations of abuse [49]. Sexual assault seems to be considered to be one of the least socially tolerated forms of abuse, although it also brings shame on the victim and her family. As such, women who suffer from sexual abuse may be more likely to seek help from non-family resources such as police or health centers, unlike with other forms of gender-based violence, where family members are the most likely source of support [52, 57]. With regard to early marriage, determining the prevalence of early marriage is not straightforward through the use of government documents, as many early marriages are registered with religious authorities and not in the official government system [55].

### Family and community environment

From the results of the literature review, several documents addressed the social determinants of SGBV and early marriage. These documents identified factors occurring at several different levels, including norms and practices within the family and community, as well as higher-level socioeconomic factors that constitute some of the root causes.

Many of the norms, attitudes, and practices that support SGBV in Jordan appear to be reinforced within families. One small, qualitative study that asked victims of SGBV to reflect on their adolescent years found that women often described patterns of abuse beginning in childhood, with gender discrimination, violence committed by fathers and brothers, and forced marriage, while some women in the study described their future husbands using rape as a means to ensure that the marriage would take place, despite the women’s wishes [46]. Another qualitative study conducted among Syrian adolescent boys and girls found that physical and psychological violence committed by family members was also a major concern [56].

Within communities, several forms of gender-based violence appears to be largely acceptable among both women and men. The 2012 JPFHS found that 70% of Jordanian women justified wife beating under at least one circumstance, rising to 84% among youth aged 15–19 years [58]. A study conducted among Syrians living in camps found that both married and unmarried men often believe that husbands are justified in beating their wives in several circumstances, including if a woman does not want children or uses family planning methods without the husband’s consent [43]. Attitudes towards the acceptability of gender-based violence appear to be reinforced in younger generations beginning at an early age. A study on community acceptance of honor killing that was conducted among 856 adolescents aged 14–16 years who were recruited from 14 schools across Jordan found that 40% of boys and 20% of girls, especially among those of the lower socioeconomic status, believe that killing a daughter, sister or wife for dishonoring the family can be justified [18].

The documents reviewed vary considerably with regard to their findings as to whether early marriage is acceptable in Jordan, with the vast majority of research having been conducted on Syrian refugees. A qualitative study conducted among Jordanians, Iraqis, Syrians, and Palestinians found that most people believed that there were certain compelling circumstances under which early marriage is acceptable, although many believed that early marriage was not generally advisable [53]. Many women who were interviewed across nationalities who were themselves married early do not think early marriage to be a positive practice, but felt forced into marrying their daughters early due to social pressure [53]. Among Jordanians, study participants overwhelmingly reported that the major contributing factor to early marriage was the fact that it was a socially-accepted tradition, and that it was believed to ensure protection for girls [53]. Among Palestinians, poverty was considered as the most important contributing factor, although providing protection for girls was also seen as an important reason for the practice [53].

Among Syrians, early marriage is thought to have been both a widespread and socially accepted practice in Syria [39, 42, 52, 53]. In Jordan, one study conducted among Syrians living in host communities found that between 20 and 45% of respondents believed early marriage to offer some benefit, and all of the study’s respondents agreed that early marriage predated the Syrian crisis as being an acceptable practice [52]. Among Syrians, the role of tradition as well as the preservation of a family’s (and woman’s) honor are some of the most commonly cited reasons [51, 53]. Young Syrian women indicate that there are clear benefits of early marriage, including obtaining more respect from the community or being able to escape a difficult family situation [52]. Norms may be shifting among youth; the results of one qualitative study conducted among both Jordanians and Syrians found that many of the youth interviewed in the study strongly rejected the practice of early marriage [42].

Among Syrian refugees in camps, five documents discussed how the social and physical environment in camp settings is one of the reasons for the increased rates of early marriage that have been observed in recent years. With the breakdown of traditional social structures and norms resulting from the conflict, as well as the high prevalence of sexual violence in camps, some families believe that early marriage is a necessity and offers a way of protecting their daughters in what is perceived as an unsafe environment, [48, 49] given the shame placed on a woman and her family if a woman loses her virginity outside of marriage, regardless of whether consent was given for the sexual encounter or not [51]. As a result, forced marriage after rape is common [48]. In a qualitative study conducted in Irbid, women indicated that early marriage occurs because fathers want someone to protect their daughters considering the poor security situation, or that they do not want to take responsibility should their daughter be raped [39]. In another study, older, unmarried girls living in camps indicated that ensuring the protection of their honor adds to the burden that they believe they place on their families [51].

Several studies also cite a number of economic and social incentives behind the increasing prevalence of early marriage among Syrians. Early marriage is thought to reduce the number of people that need to be provided for in a family [53], while also serving as a source of income through the customary transfer of money and/or wealth from the groom’s family to the bride’s family (in Arabic, *Al Mahr* or المهر) and gaining a son-in-law [49]. In Syria, the exchange of goods or money for a bride is a common practice. In a study conducted among Syrian refugees living outside of camps, 6.7% of those surveyed indicated that the resources they receive for their daughters is one of their top three sources of income [52]. Furthermore, for some, the opportunity to gain access to certain social services offers an incentive to engage in early marriage. In some cases, early marriage may offer families a way out of the camp if their daughter marries a non-Syrian, thereby serving as means for both the woman and her family to obtain greater benefits, state-support, and security in an unknown environment [52, 53]. The official policy of the Jordanian government is that Syrians can legally move into host communities when sponsored by a Jordanian, which has caused concern that as Jordan’s economic situation worsens, early marriages between Syrians and non-Syrians may increase [43]. One study that interviewed key informants in camps also echoed this fear, indicating that men from other countries have been found in camps looking for young brides [39]. This could create a predatory environment for young women in those settings. Qualitative data indicates that some Syrian families are becoming increasingly concerned about the interest of foreign men in Syrian girls, and have begun to reject marriage proposals from non-Syrian men, as they are thought to be disrespectful to their daughters [43].

### Service delivery environment

No studies were found that focus on the health service delivery environment specifically related to SGBV for Jordanians, Iraqis, or Palestinians. Knowledge of service availability for survivors of SGBV appears to be extremely low among Syrians. The results of one survey indicate that a vast majority (83%) of Syrian refugees that participated in the study were not aware of any SGBV services in their community and that very few women would turn to a health clinic as a first choice to obtain services in the case of sexual violence [52]. In one study conducted among health service providers in Syrian refugee camps, results indicated that they generally had very limited past experience with sexual assault and low confidence in responding to such cases [50].

Documents pertaining to several programs sponsored by international and local NGOs were retrieved. A summary of programs obtained from the literature and program documentation is included in Table 2. The majority of them focused on awareness-raising initiatives related to SGBV and early marriage [40, 60–62], including mass media initiatives [33]. The United Nations Population Fund (UNFPA) provides case management, psychosocial support and legal services for survivors of SGBV in camp settings [49]. No documentation was found from programs outside of the camp setting focused on survivors among Jordanian or refugee youth populations.

**Table 2:** Summary of programs focused on sexual and gender-based violence (SGBV) in Jordan since 2008 retrieved in literature review

*16 Days of Activism Against Gender-Based Violence* [33] UN Women organizes an annual global campaign entitled “16 Days of Activism Against Gender-Based Violence. In 2015, several Jordanian civil society organizations participated and Princess Basma Bint Talal, supported the campaign. *Arab Women Organization of Jordan* (AWO) AWO is engaged in several initiatives to promote women’s participation in policy and civil society, as well as advocating for increased gender-equitable policies [59]. *Safe Spaces* [49] UNFPA-supported safe spaces includes case management, psychosocial support, and legal services for survivors of GBV. In Zaatari Camp, UNFPA runs OASIS space in conjunction with UN Women. Safe Spaces are closely aligned with SRH services to facilitate care and referral. *Amani*Supported by UN Agencies, including UNFPA, UNHCR, and UNICEF, Amani is an information campaign that focuses on child protection and gender based violence, and recognizes early marriage as a form of sexual and gender-based violence. The campaign provides communities with informational material to distribute that targets both adults and children through the story of a girl named “Amani” and her family. Communities distribute the messages through learning spaces, local radio, health clinics, etc. [40] *Arab Women Speak Out (Irbid: 2011, Zarqa: 2009–2010)* [60, 61] The Arab Women Speak Out is a program implemented by the Jordan Health Communication Partnership (funded by USAID) that consists of two parts: 1) a training encourages participants to explore SRH subjects through participatory exercises and presentations and 2) an information dissemination program that uses social networks to distribute flash cards with messages about reproductive health, including early marriage. The results of the second phase in Irbid include a focus on early marriage. At baseline, 85% of participants disagreed with the statement that “early marriage is a good basis for a happy and stable life.” At endline, over 90% of all participants reported discouraging their daughters or nieces from marrying before the age of 18 and their sons or nephews before the age of 22 [44]. *Emergency Assistance for Refugees and Host Communities affected by the Syrian Crisis in Jordan (2017)* [62] This activity was funded by DfID, and was implemented by CARE International and the Jordan Women’s Union. In 2017, 54 awareness raising sessions were conducted for 1620 women and adolescents, with an average 30 participants per session, at CARE centers in Amman, Azraq, Irbid, Mafraq and Zarqa. Topics discussed in the awareness-raising sessions included: reproductive health, the psychological and physical effects of violence, and sexual violence and mechanisms of protection against sexual violence. Under this project, at JWU, CARE also conducted a training of trainers (ToT) for 10 CARE staff, to enhance their knowledge and support in psychosocial activities, particularly on the topic of SRHR. The majority of respondents reported that their participation has a direct effect on enhancing their social wellbeing (77%), it increased self-esteem (74%), enhanced stress relief (61%), increased their information and skills (54%), enhanced their negotiation and communication skills (45%), increased their mobility in public space (36%), and enhanced their emotional regulation (36%). In addition, 30% of crisis-affected population (especially women and adolescent girls of reproductive age) reported accessing at least one sexual and reproductive health service through support by CARE and/or its partners. *My Vision for the Future: an adolescent and youth focused assessment on GBV and SRHR (2018)* [62] This project is an ongoing research activity conducted by CARE International in Partnership with Plan International. The purpose of this assessment is to support the delivery of programming for the prevention and response to GBV among adolescents and youth in Jordan. The assessment aims to 1) describe the existing evidence base for interventions to address GBV as it affects female and male adolescents and youth in Jordan and 2) Identifies solutions for preventing and respond to GBV in Jordan to be implemented with adolescents and youth. Additionally, this study will attempt to identify means to support adolescent and youth survivors of sexual violence and young married women through sexual and reproduction health (SRH) services.	

Within the domain of health services, FGD emphasized that the Ministry of Health has integrated violence screening into reproductive health services. Specifically, health providers at maternal/child health and family planning centers are required to screen pregnant and post-partum women for violence during and after giving birth. The reporting of data on SGBV at health facilities remains uneven and there was a lack of agreement between FGD participants on the reporting requirements. While one Jordanian Government official mentioned that “each doctor will ask about violence to avoid legal liability;” another official said that “not all centers report violence, unless they were trained and have a family protection committee.” An additional concern highlighted by the Government of Jordan representatives is that data are not specifically reported on youth populations at the national level.

Focus group participants also highlighted the need for improved programming through the education sector focused promoting knowledge related to SGBV from a rights-based perspective. Specifically, participants highlighted the power of programs focused on educating youth about their bodies and their rights in preventing sexual violence. For example, one participant from an NGO said “in my work we see many rape and statutory rape cases by family members. If the child knew that his\her body is private, he\she would be able to report it if any danger [ous] situation occurred, or if anyone tries to touch him\her in inappropriate places, but unfortunately children think it is normal,” while another NGO participant mentioned that there have been success stories of children reporting violence “when [they] hear messages in school that their body shouldn’t be touched by anyone.”

### Policy and legal environment

Few documents were found that relate to the policy environment surrounding SGBV among youth in Jordan. While one of the documents retrieved emphasizes that there is no policy in Jordan that provides guidance in cases of SGBV or care for survivors [28], according to the National Council for Family Affairs (NCFA) there exists a “National Framework for Family Protection against Violence” which includes definitions of terms related to violence against women and children and laws pertaining to family protection [63]. Additionally, the Ministry of Health and UNFPA have developed guidelines, trainings, and distributed post-rape kits in camp settings, but according to a health-sector assessment, the quality of care for survivors of sexual violence still needs significant improvement [64].

Despite the lack of clear documentation retrieved from the literature review, focus group participants emphasized that family protection is a national priority in Jordan. The NCFA leads a national team focused on domestic violence. One representative from a UN Agency emphasized that this is one of the areas of success, in that “[it] is one of the more powerful teams before the law, and it has its own system and authority to make sure programs are executed.” Participants highlighted that the NCFA recently approved a national strategy on domestic violence and has developed detailed implementation procedures for institutions on SGBV programming, which includes a training curriculum for health and social workers. Currently, there are approximately 10 family counseling offices located across Jordan that focus on preventing and responding to cases of family violence. Current activities focus on awareness-raising about SGBV that are integrated into ongoing community outreach activities, including integration into early childhood programs to prevent violence against children. According to a representative from the Government of Jordan, whether community outreach activities in schools include SGBV is up to the community health committees and their review of existing data: “If a local community says we have a problem in this area, then education and awareness programs are implemented, however if the community doesn’t mention it as a problem that they face, it doesn’t take priority.”

With regard to honor killing, there remains legal justification for honor killings in Jordan [37, 38]. Although Article 98 of the Jordanian Penal Code allows for reduced sentences for perpetrators of honor killings if there are mitigating circumstances, Jordanian courts have recently begun dismissing claims of mitigating circumstances and imposing longer sentences [18, 33]. Mitigating circumstances include a man’s “fit of fury” or a woman’s “wrongful and dangerous act,” including adultery [33]. In the case of adultery, a man is exempt from any penalty [18]. The defense of mitigating circumstances is not available if the victim is under 18 years of age [33]. Prior to judiciary reforms pertaining to honor killings in 2001, the average sentence ranged between three months and two years [18].

The documents reviewed highlighted several ways in which the policy and legal environment could be strengthened to protect girls from early marriage. Early marriage remains legal in both Jordan and in Syria. In Jordan, the minimum age of marriage is 18 years for both girls and boys, but girls can marry as young as 15 years with approval from a Shari’a court [43, 48]. Despite legal restrictions on the conditions under which minors are allowed to marry, the fact that early marriage remains relatively common in Jordan indicates that the marriages are commonly approved despite the restrictions in the law [48]. In Syria, the legal age of marriage for girls is younger than in Jordan (16 years), but girls as young as 13 can marry with the approval of Shari’a courts [43, 53]. Islamic religious officials have issued some fatwas (religious rulings or decrees), against early marriage; however, they do not appear to have had an effect on limiting the practice [28].

Focus group participants added that with regard to early marriage, the NCFA also heads the National Commission to Reduce Early Marriage, which is focused on advocating for and approving policies related to early marriage and has put into place an action plan to continue efforts to limit early marriage in Jordan. As the National Commission focuses their efforts at the policy level rather than on service delivery, their activities center on advocacy, such as expressing the needs of women and girls to policymakers and raising awareness about the legal status of and concerns associated with early marriage in communities among parents. Participants mentioned the Commission’s 16-day campaign to limit early marriage that was implemented in conjunction with the Higher Population Council. The Campaign focused on “raising awareness about the negative impact early marriage has on girls’ mental, physical, and reproductive health.” Additionally, stakeholders indicated that a policy paper will soon be published on early marriage that was developed in a participatory consultative process that included the Chief Justice Department and lawmakers, and efforts are also currently underway to track projects and activities related to limiting early marriage.

From a legal perspective, FGD participants indicated that the current laws are easy to work around and contain many loopholes – thus highlighting the need for stronger laws that restrict early marriage even further that are coupled with improved enforcement. A participant from a UN Agency emphasized that, “there must be a certain process, which has clear guidelines that can’t be crossed, that we can rely on, but what happens now is the following … some fathers will bring their daughters to the court and say ‘I want you to approve my daughter’s marriage’ … and it will be approved, without any study on the benefit to the girl.” Another participant from an NGO expressed a similar observation, “Even though they apply the law, you can still get approval for marriage under the age of 18. What we have to do is increase the restrictions [on the conditions that allow for early marriage].” Some participants indicated that early marriage is unlikely to be eradicated, given that many populations in Jordan believe it continues to serve an important purpose of protecting young women.

Participants also emphasized several important challenges in enforcing policy and laws related to early marriage. From a social perspective, they indicated that marriage under the age of 18 years has become the norm in many rural communities, especially among Syrian refugees. Participants emphasized that while many believe early marriage to be primarily a problem among Syrians, it is becoming increasingly common among Jordanians in rural areas as well, though data are limited in this area. Programs focused on transforming social norms related to early marriage are necessary in order to shift public opinion. Several participants indicated that “spreading awareness about the effects of child marriage physically, psychologically, and socially through religious channels” may be one way to bring about community support, and engaging youth through internet-based approaches, social media, and influential figures could also be promising. Other participants also mentioned that generating evidence for the negative consequences of child marriage could be effective, including divorce and maternal/neonatal mortality. One NGO participant mentioned that a driver behind early marriage is early pregnancy, “those who attempt early marriage [for their daughters] also have in their mind immediate pregnancy,” thus raising awareness of the dangers of early pregnancy may help garner support.

Given the gaps in the legal environment, several non-governmental organizations have engaged in the policy sphere to coordinate activities aimed to prevent early marriage. In 2013, the Sexual and Gender-Based Violence Sub-working group established the Forced and Early Marriage Task Force to serve as a platform to exchange information, provide technical support, develop joint actions to address the issue of forced and early marriage, build capacity of stakeholders and develop joint actions and strategies [53]. Additionally, the United Nations High Commission for Refugees is working in collaboration with the Government of Jordan to improve the personal status law which contains legislation on the legal age of marriage [51].

A few documents expressed concern with regard to the way that the early marriage has become a focus at the policy-level through advocacy efforts and by way of the media. In one study, a key informant believed that advocacy efforts on early marriage have reduced the capacity of NGOs to respond to issues related to early marriage and other forms of SGBV because of backlash and increased sensitivity to the issue, especially among Syrians [39]. Additionally, the media attention to early marriage is believed by some program managers to have negatively influenced how Jordanians view Syrians, which has in turn, increased sexual assault and harassment of Syrian girls by individuals of other nationalities [51].

Participants in the FGDs also echoed similar concerns. One participant cautioned that there has been some backlash to external efforts geared towards shifting social norms around early marriage. Participants emphasized that any work in this domain must be careful to use terminology that comes from within communities, rather than through phrasing that implies an external agenda. For example, even the term “early marriage” is often problematic as community members do not fully understand it given that it is a normative cultural practice and therefore, they may reject programs focusing on it as being insensitive to their local customs. A participant from a UN Agency mentioned that, for example, “[the term] child marriage is not clear to them when we talk about it, even us in the institutions use the term in the wrong way. We are still a tribal society and the presenter should have acceptance from the community.”

## Discussion

The results of this study indicate that norms, attitudes, and policies that support practices related to SGBV and child marriage are deeply entrenched within families and communities, are reinforced by the restrictive gender norms that limit the freedoms and opportunities afforded to girls, and are codified in the legal frameworks of both government and religious institutions that allow space for these practices to continue. At the individual level, many of the drivers of SGBV in Jordan appear to mirror the surrounding social, economic, and political context that reinforces the low status of women. Supporting women and girls to challenge and overcome the discriminatory norms that limit their participation in economic, social and political life such as by encouraging financial independence among women and supporting them to develop life skills may shift the way that women are viewed in society. Further, ensuring that women have information about where to seek help and report abuse remains critical, especially as women begin to challenge traditional roles.

At the community level, the social environment appears to largely reinforce harmful norms that put young people at risk of violence, while the worsening economic conditions and the lack of educational opportunities within communities create an environment where young people are disempowered and certain forms of violence, such as early marriage, are thought to be protective [65]. This may be especially true in refugee communities where limited economic opportunities combined with the impact of war, trauma and ongoing displacement to create safety concerns for both young women and men. Within communities, this study highlighted considerable gaps in the social, health and educational resources that that are available to youth, but especially young women and girls. Strengthening such services could be an opportunity to promote and reinforce gender equity and respectful interpersonal relationships. Furthermore, given that the enforcement of laws related to many areas of SGBV remains weak in Jordan at the national level, informal community groups may represent a particularly important opportunity to support women in reporting abuse while putting pressure on communities to stop granting permission for early marriage [48, 66]. Table 3 provides a synthesis of key programmatic and policy-related recommendations that emerged from the literature and FGDs according to the different levels of the social ecological model.

**Table 3:** Summary of Key Lessons Learned and Opportunities for Intervention to improve Sexual and Gender-based Violence and Early Marriage Among Youth in Jordan

**Individual level** • *Ensure that youth have access to sexual and reproductive health information and services, including emergency contraception and post-rape care.* • *Encourage young girls to stay in school and promote life-skills and livelihoods training and financial independence* [46, 52]. When young girls stay in school and succeed in their studies, they are less likely to be seen by their families and communities as ready for marriage [48]. Additionally, girls that are able to generate income may be seen as more valuable by their families [48]. • *Ensure that youth have access to quality SRHR information and services.* Girls that are informed about their reproductive health may be more able to refuse early marriage [48]. • *Engage men and boys* to support transformation of harmful gender norms. **Family and community levels** • *Transform gender norms early that lead to discrimination against girls and women and early marriage.* Support teachers to eliminate discrimination in the classroom, and promote gender equity, respectful inter-personal relationships, and peaceful conflict resolution through the mandatory curriculum in schools [33]. • *Ensure schools have adequately trained social workers* to support youth who experience gender-based violence in their homes. Build community protection committees and link them to other existing structures such as parent-teacher associations, school councils, child friendly spaces [49]. • *Engage communities in addressing early marriage* by involving women, community and religious leaders, and educating them about the risks of early marriage [48, 49] Invest in informal policy and regulatory bodies within communities so that they are less willing to grant permission for early marriages [48, 66]. ***Policy and Service Delivery level*** • *Integrate mental health services* with SRHR services, and ensure access to quality services for sexual and gender-based violence for women and youth [28, 49]. Ensure that the health providers actively screen for violence as part of routine service provision [36]. Mobile services should also be strengthened to reach rural areas [52]. • *Support legal reform* that empowers women to come forward by providing them with access to legal resources [52] and holds perpetrators of GBV and honor killing accountable. • *Strengthen laws that prohibit early marriage*, [28] including increasing the minimum age at which discretionary permission to marry can be granted by a shari‘a court and issuing more precise instructions with regard to the application of the special permissions to marry below the age of 18 [53]. • *Improve the enforcement of laws on early marriage* so that law is systematically applied [36, 48].	

The results of this study also highlight that there is little published in the formal literature on SGBV among youth in Jordan and research gaps remain that would help to better inform both program and policy. More in-depth research on the prevalence and the determinants of SGBV, honor killing, and early marriage across Jordan is needed to identify the youth and children most at risk and to improve the salience of programs in prevention and response [48]. Given the high level of acceptance of SGBV and honor killing, a deeper understanding of how norms are transmitted within Jordanian society could also help intervention efforts. Additional research conducted among Jordanians and other vulnerable populations is needed, including refugees living outside of camp settings and boys. Some research in the region has indicated that boys, especially those who are vulnerable because of their refugee status, may face increased risk of being victims of sexual violence, but very little data exists in this domain given its social sensitivity. The results of only one study included in this review highlighted this issue. Furthermore, the fact that it was not discussed in the FGDs may highlight the limited awareness about this issue among key stakeholders.

As early marriage is not as sensitive of a topic in Jordan as other forms of SGBV, research may be more easily conducted on this topic through existing arenas such as safe spaces, registration centers, monitoring programs, etc. than on other domains of violence [56]. Research is limited on the sexual, reproductive, maternal, and mental health consequences of early marriage and the intersection between these dimensions [67]. A better understanding of these consequences may help to better support young women with the range of services they need, while also providing additional data that could be useful in both awareness raising and advocacy efforts.

With regard to health services for survivors of SGBV and early marriage, research is needed to better understand how youth, and other affected populations, perceive existing services and how they could be improved. There is currently a limited understanding of the gaps in the existing service delivery infrastructure available to support survivors of SGBV, and more research is needed to assess providers’ competence and facility in providing services to survivors of SGBV. While research to improve the service delivery environment is indeed a priority, more research is also needed on the barriers faced by adolescents in accessing and utilizing existing services, considering the social and cultural environment.

The results of this analysis presented in this paper may be subject to several important limitations. Even though every attempt was made to be exhaustive, some documents may have been arbitrarily excluded. This may have occurred for several reasons, including that they did not appear in the search results or did not focus on youth specifically. This concern may be especially true with regard to the gray literature search. A challenge in Jordan, as in many countries, is that much of the programmatic literature goes unpublished and is not widely disseminated. While a strength of this study is that extensive efforts were undertaken in order to retrieve as many relevant, gray literature documents as possible, some may not have been retrieved, as many organizations’ reports remain unpublished, or organizations may not have responded to queries. Furthermore, while the FGDs provided robust data on a variety of topics, some participants may have been hesitant to be critical of programs or policy, given that they were representing their organizations.

## Conclusions

The purpose of this landscape assessment was to consolidate and synthesize the results and lessons learned from across the research, programmatic, and policy domains pertinent to youth and SGBV and early marriage in Jordan while taking into account the nested layers of contextual influence. By examining SGBV and early marriage from multiple levels, this review analyzes these phenomena as being part of a social ecological system, whereby determinants interact across personal, situational, and sociocultural factors [7], and offers a unique perspective on programmatic experience and research in Jordan. As the Middle East continues to face instability, and as a result, many countries across the region continue to struggle with the urgent health needs of large refugee and youth populations, this review provides valuable insight relevant to research, programs, and policy across the region.

## Data Availability

The datasets used and/or analyzed during the current study are available from the corresponding author on reasonable request.

## Abbreviations

FGD: focus group discussion
IRB: Institutional review board
JPFHS: Jordan Population and Family Health Survey
NCFA: National Council for Family Affairs
NGO: Non-governmental organization
SGBV: sexual- and gender-based violence
UNFPA: United Nations Population Fund
UN: United Nations
